# Epidemiological role of dogs since the human leishmaniosis outbreak in Madrid

**DOI:** 10.1186/s13071-017-2147-z

**Published:** 2017-04-26

**Authors:** Guadalupe Miró, Aurora Müller, Ana Montoya, Rocía Checa, Valentina Marino, Eloy Marino, Fernando Fuster, Cristina Escacena, Miguel Angel Descalzo, Rosa Gálvez

**Affiliations:** 1 Grupo de Investigación Epicontrol-Carnívoros, Departamento de Sanidad Animal, Facultad de Veterinaria, Universidad Complutense de Madrid, Avda. Puerta de Hierro s/n, 28040 Madrid, Spain; 2 Dirección General de Salud Pública, Consejería de Sanidad, Comunidad de Madrid, c/Ronda de Segovia 52, 1ª planta, 28005 Madrid, Spain; 3 Unidad de Investigación, Fundación Piel Sana Academia Española de Dermatología y Venereología, Calle Ferraz, 100, Madrid, Spain

**Keywords:** Canine leishmaniosis, Dog, Human leishmaniosis, *Leishmania infantum*, Madrid, Outbreak

## Abstract

**Background:**

Canine leishmaniosis (CanL) has been in the spotlight since the 2009 outbreak of human leishmaniosis in Madrid. In the framework of the Leishmaniosis Surveillance Programme set up in Madrid, this study examines *Leishmania*-specific seroprevalences in stray dogs for the outbreak area and rest of the Madrid region over the period spanning from the outbreak to the present (2009–2016). These data are of interest because stray dogs could be sentinels for disease surveillance in endemic areas. Since 2011, we have also been monitoring owned dogs in the outbreak area.

**Methods:**

Over the study period, *Leishmania infantum* seroprevalence was determined in 2,123 stray dogs from the outbreak and non-outbreak areas. A serological study was also performed for owned dogs in the outbreak area: high-risk dogs such as hunting or farm dogs (*n* = 1,722) and pets (*n* = 1372). All dogs were examined and blood was collected. The variables recorded for each animal were: breed, age, sex, and clinical history indicating if the animal was healthy or clinically suspected of having any disease, and if they showed a clinical picture compatible with CanL.

**Results:**

Seroprevalences of *L. infantum* in stray dogs were similar in the two areas examined: 4.7% (20 out of 346) in the outbreak area and 5.4% (96 out of 1,777) in the remaining Madrid region (*χ* ^2^ = 0.080, *P* = 0.777). A significant association was found between seroprevalence and age (*z* = -6.319; *P* < 0.001). Seroprevalence in owned dogs in the outbreak area was 2.1% in high-risk dogs (37 out of 1,722) and 1.2% in pets (17 out of 1,372) (*χ* ^2^ = 3.561, *P* = 0.0591).

**Conclusions:**

Both stray and owned dogs do not seem to play an important role in maintaining the transmission cycle of *L. infantum* in the Madrid outbreak area. The stable seroprevalence of infection observed in sentinel dogs suggests the good clinical management and prevention of CanL by local practitioners in owned dogs.

## Background

Leishmaniosis caused by *Leishmania infantum* has been in the spotlight since in 2009 the largest human leishmaniosis (HL) outbreak in Europe affected the south-west area of the Madrid region (Spain), mainly the districts of Fuenlabrada, Leganés, Getafe and Humanes [1]. Close cooperation between the local government and scientists has been key to ensure prompt response strategies to manage this outbreak [1]. A multifaceted approach by physicians, public health professionals, entomologists and veterinarians was required to address all determinants involved in this disease under the One Health approach [2].

Leishmaniosis is a zoonotic endemic disease widespread in the Mediterranean basin and transmitted to humans and animals by blood-sucking phlebotomine sand flies [3]. Dogs are considered the main reservoir for human infection by the protozoan parasite *L. infantum*. However, in the past decade wild animals such as lagomorphs and canids other than dogs have been attributed an important role in the transmission cycle [4–8]. In Spain, the disease is transmitted by the sand flies *Phlebotomus perniciosus* and *P. ariasi* [9–11]; *P. perniciosus* is the main vector in the Madrid region [12–14].

From 1991 to 2004, the incidence of HL in this region was established at 1.12 cases/100,000 inhabitants per year [15]. The sporadic number of human cases was accounted for by infants (aged 0–4 years) and immunocompromised adults with human immunodeficiency virus (HIV) co-infection. The introduction of highly active antiretroviral therapy in 1997 cause the reduction of these co-infections [16, 17]. Since mid-2009, the incidence of HL increased abruptly in the south-west region of Madrid also among immunocompetent adults, most HL cases being cutaneous leishmaniosis forms [1]. The global incidence reported from mid-2009 to December 2016 was 16.27 cases/100,000 inhabitants including 702 new cases. The annual incidence of HL has drastically fallen in the last two years, 2015 and 2016 (data provided by the Division of Epidemiology, Health Promotion and Prevention Subdirectorate, Primary Care Directorate, Madrid, Spain).

Environmental changes provoked by the construction of green parks in a large urban setting have led to an increased density of the newly incriminated lagomorph reservoir (mainly hares) that sustains a large sand fly population. Entomological studies have identified *P. perniciosus* as the predominant species in this area [18], where *P. perniciosus* shows a high prevalence of *L. infantum* by PCR (58.5%) [19]. Studies of blood meals of *P. perniciosus* captured in this area have revealed a preference for hares [19]. Xenodiagnostic studies have shown the ability of hares and rabbits (*Oryctolagus cuniculus*) seropositive for *L. infantum* to infect *P. perniciosus* [6, 7].

Other reservoirs such as cats in the area affected by the HL outbreak have also been examined, and seroprevalences of *L. infantum* in the range 3.2–9.3% (cut-off ≥ 1:100 or ≥ 1:50, respectively) have been reported [20, 21]. These seroprevalences have not substantially changed since the first studies conducted in Madrid before the outbreak [22, 23]. Hence, cats have probably not played a crucial role as a reservoir for *L. infantum* infection since the outbreak [20].

So far, few studies have examined the role of dogs in the HL outbreak. In 2011 and 2012, a canine serological survey in the affected municipal districts reported surprisingly low seroprevalence levels of 1.0–3.6% in dogs [1] compared to seroprevalence data obtained over the past 15 years in the Madrid region, which have ranged from 6.4 to 8.1% [24–26].

To elucidate the epidemiological role of dogs in the Madrid outbreak, this study sought to identify possible differences in *L. infantum* seroprevalence between stray dogs in areas affected by the HL outbreak and non-affected areas of the Madrid region. The data analysed correspond to the period from the outbreak to the present (2009–2016). Possible correlating factors were also examined. To complete this information, seroprevalences in owned dog in the outbreak area have been monitored from 2011 to 2016 making a distinction between high-risk owned dogs and pets.

## Methods

### Study area and dog populations surveyed

The study area (Madrid Autonomous Community) has 11 established health areas. A health area is defined as the largest area grouping primary and specialized health care services. Health care areas vary in size according to geographical, cultural, and epidemiological factors along with available infrastructure.

Classification schemes for dog populations were based on their lifestyles [27, 28]. The animals surveyed (*n* = 5217) were divided into the following three dog populations:

- Stray dogs (*n* = 2,123). Dogs abandoned in the Madrid region living in a municipal animal shelter until their adoption.
- Owned dogs: (i) High-risk owned dogs (*n* = 1,722). These dogs lived in animal shelters in the outbreak municipalities and were assumed to carry a high risk of exposure to sand fly bites because of their outdoor lifestyle. In addition, the owners of these dogs reported a non-regular use of prophylactic measures like topical insecticides. The animal shelters surveyed included hunting dogs living in kennels, pets in boarding shelters and dogs living on farms. (ii) Pets (*n* = 1,372). Owned dogs enrolled during the annual compulsory rabies vaccination programme in the municipalities affected by the outbreak. These dogs were assumed to be better cared for and thus at lower risk of infection.

**Fig. 1:**
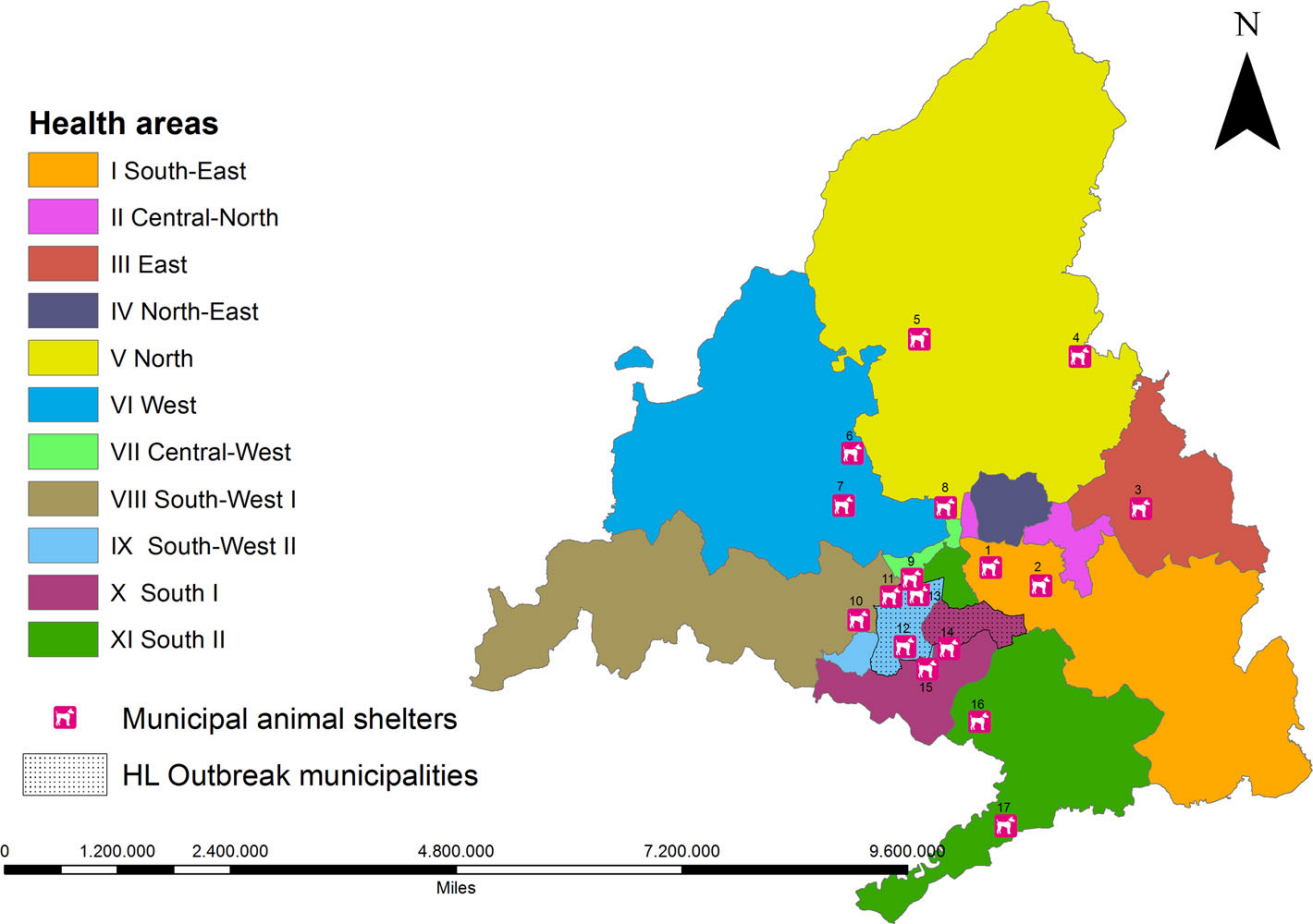
Municipal animal shelters (*n* = 17) in the corresponding health areas in Madrid, Spain

To survey the stray dog population, each of Madrid health areas was represented by at least one municipal animal shelter with the exception of the three smaller areas which were surveyed as one. The owned dog populations were only surveyed in the outbreak municipalities (Fig. 1).

### Serological study

This study was carried out in accordance with international guidelines for the Care and Use of Experimental Animals and Spanish Legislation (RD 53/2013). Each dog in each of the three populations was subjected to a clinical examination and blood was collected by cephalic venipuncture. Serum was used for antibody testing. Antibodies against *L. infantum* were detected by the indirect immunofluorescence anti-body test (IFAT). IFAT for anti-*Leishmania*-specific immunoglobulin G (IgG) antibodies was performed as described previously [29] using a cut-off of 1:100. Seroprevalence was calculated as the percentage of dogs testing positive for anti-*L. infantum* antibodies.

### Leishmaniosis surveillance programme

In 1996 (before the HL outbreak), a Leishmaniosis Surveillance Programme for stray dogs was set up in the Madrid region. This programme detects risk factors, monitors infection trends, and when necessary, takes different courses of action. Every year, the seroprevalence of *L. infantum* infection among stray dogs at municipal animal shelters (*n* = 17) was determined twice, in spring and autumn. These collaborating animal shelters in the Madrid region are distributed across nine health areas (Fig. 1).

*Leishmania infantum* seroprevalences were determined in 2,123 stray dogs from 2009 to 2016. The variables recorded in each animal were breed, age (minimum age was 6 months), sex, and clinical history.

### Serological surveillance in the outbreak area

In 2011 after the HL outbreak onset, a Leishmaniosis Surveillance Programme was also established for owned dogs in the outbreak area. Between 2011 and 2016, the seroprevalence of *L. infantum* infection was determined in 1,722 high-risk owned dogs and between 2011 and 2012 in 1,372 pets. Our serological surveillance programme for owned dogs started in 2011 and is still ongoing for high-risk owned dogs but not for pets since seroprevalences in pets were extremely low (Table 1).

**Table 1:** *Leishmania infantum* infection seroprevalence recorded in owned dogs in the outbreak area

Year	High-risk owned dogs	Pets
	Positive/Total (%)	Positive/Total (%)
2011	7/196 (3.6)	8/811 (1.0)
2012	10/502 (2.0)	9/561 (1.6)
2013	10/415 (2.4)	–
2014	3/209 (1.4)	–
2015	4/200 (2.0)	–
2016	3/200 (1.5)	–
Total^a^	37/1,722 (2.1)	17/1,372 (1.2)

^a^Chi-square without Yates correction (*χ* ^2^ = 3.561, *df* =1). The two-tailed *P*-value was 0.0591 and thus not significant (significance was set at *P* ≤ 0.01)

### Statistical analysis

The Chi-square and Mann–Whitney U tests were used to identify significant associations between *L. infantum* seroprevalence and age, sex or breed. Seroprevalences were calculated separately for the outbreak area and non-outbreak area both yearly and globally. The Chi-square test was used to determine *L. infantum* seroprevalence differences between the two zones. Significance was set at *P* ≤ 0.01. All statistical tests were performed using the SPSS 22 package (SPSS Inc., Chicago, IL, USA).

## Results

### Leishmaniosis surveillance programme: stray dogs

The stray dog population was well-balanced in terms of sex (female: 958 dogs, 45.6%; male: 1,145 dogs: 54.4%), breed (mixed: 1,333, 62.9%; purebred: 785 dogs, 37.1%) and number of dogs examined each year (2009: 206 dogs, 9.7%; 2010: 203 dogs, 9.6%; 2011: 210 dogs, 9.9%; 2012: 302 dogs, 14.2%; 2013: 299 dogs, 14.1%; 2014: 303 dogs, 14.3%; 2015: 299 dogs, 14.1%; 2016: 301 dogs, 14.2%). Moreover, stray dogs were captured in a proportioned manner from the nine health areas surveyed (I: 250 dogs, 13.7%; III: 148 dogs, 8.1%; V: 553 dogs, 30.4%; VI: 255 dogs, 14.0%; VII: 222 dogs, 12.2%; VIII: 147 dogs, 8.1%; IX: 151 dogs, 8.3%; X: 195 dogs, 10.7% and XI: 202 dogs, 11.1%).

Specific anti-*Leishmania* IgG antibodies (antibody titre ≥ 1/100) were detected in 20 of 346 dogs from the outbreak health areas IX and X, and in 96 of 1,777 dogs from the non-outbreak areas, rendering seroprevalences of 4.7 and 5.4%, respectively.

On the basis of a physical examination: 77 of 116 infected dogs were described as clinically healthy (66.4%) and 39 out of 116 (33.6%) showed clinical signs consistent with CanL. The clinical signs observed in our study were among those commonly observed in dogs infected with *L. infantum*. Cutaneous lesions (mainly exfoliative dermatitis and/or ulcerative forms), weight loss, and generalized lymphadenomegaly were the most common clinical signs found.

We detected no differences in terms of sex (*χ* ^2^ = 0.140, *P* = 0.709) or breed (*χ* ^2^ = 0.980, *P* = 0.322) between dogs testing seropositive for *L. infantum* infection and those testing seronegative. In contrast, the mean age of seropositive dogs was 36 months compared to 24 months for seronegative dogs (*z* = −6.319, *P* < 0.001).

**Fig. 2:**
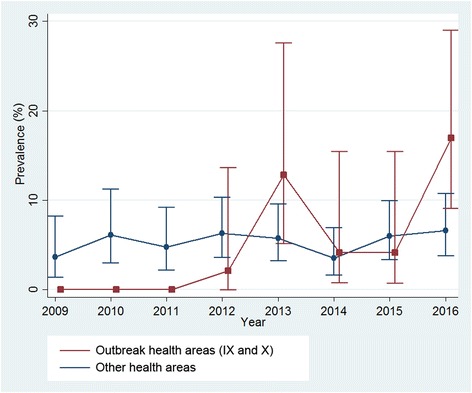
*Leishmania infantum* infection seroprevalence among stray dogs by year and area (outbreak health areas IX and X *vs* other health areas)

No significant differences between the two zones (outbreak, non-outbreak) were detected in seroprevalences both yearly: 2009 (*χ* ^2^ = 1.630, *P* = 0.202); 2010 (*χ* ^2^ = 2.501, *P* = 0.114); 2011 (*χ* ^2^ = 1.100, *P* = 0.294); 2012 (*χ* ^2^ = 1.351, *P* = 0.245); 2013 (*χ* ^2^ = 2.702, *P* = 0.100); 2014 (*χ* ^2^ = 0.047, *P* = 0.829); 2015 (*χ* ^2^ = 0.246, *P* = 0.620); 2016 (*χ* ^2^ = 6.423, *P* = 0.011); or globally (*χ* ^2^ = 0.080, *P* = 0.777) (Fig. 2).

### Serological surveillance of owned dogs

Specific anti-*Leishmania* IgG antibodies (antibody titre ≥ 1/100) were detected in 37 of the 1,722 high-risk owned dogs and in 17 of the 1,372 pets to give seroprevalences of 2.1 and 1.2%, respectively. Over the years surveyed, the seroprevalence observed in high-risk owned dogs ranged from 1.4 to 3.6% and in pets ranged from 1.0 to 1.6% (Table 1).

## Discussion

Public health concerns over *L. infantum* infection are related to its zoonotic nature and dogs are considered to be the main reservoir. In the Madrid outbreak area, we detected no increase in *L. infantum* infection among dogs over the period spanning from the outbreak to the present (2009–2016).

The construction of large green parks in the districts affected by the outbreak has led to strong readjustments in the transmission cycle of *L. infantum* [1, 30]. Changing scenarios promote alterations in the dynamic balance between host, vector, and pathogen populations that interact spatially [31]. These events are causing the redistribution of pathogens adapted to new hosts and environments, and are likely to become a public health issue of major relevance [32]. In the framework of this outbreak, there was a reported increased density of lagomorphs, both hares and rabbits [21, 33], and of sand fly populations breeding in the same environment [18, 19]. These newly incriminated wild reservoirs are able to sustain a sylvatic cycle as indicated by the findings of xenodiagnosis and molecular studies [6, 7, 19]. This new transmission cycle breaks conventional epidemiology schemes recognised for this zoonotic agent [34].

**Fig. 3:**
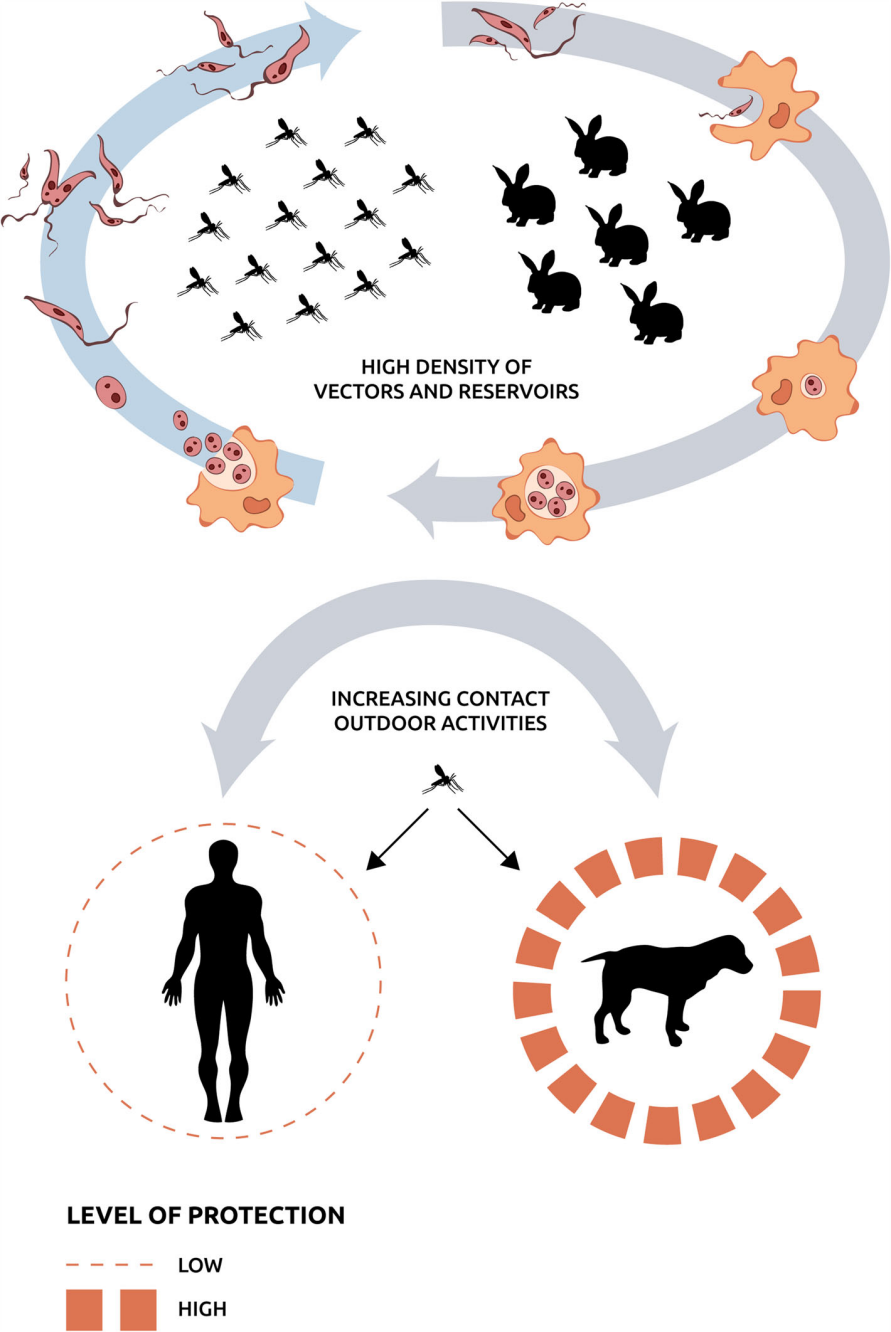
Diagram illustrating the sylvatic cycle sustained by high density lagomorph and sand fly populations and its connexion with the domestic cycle through outdoor activities conducted by dogs and humans

The existence of a sylvatic cycle independent of the domestic cycle explains why lagomorphs, and not dogs, are the driving key element in this outbreak. Several surveys conducted among persons living in the outbreak area have revealed that dogs as well as humans are at risk of *P. perniciosus* bites in parks where the sylvatic cycle is established [19, 35]. Seroprevalences of *L. infantum* infection recorded in owned dogs have remained stable probably because these animals are well protected. On the contrary, we as humans are not as accustomed to the use of preventative measures against sand fly bites (data provided by the Division of Epidemiology, Health Promotion and Prevention Subdirectorate, Primary Care Directorate, Madrid, Spain) (Fig. 3).

This lower *Leishmania* seroprevalence detected in the outbreak area in both stray and owned dogs is likely attributable to the long-standing measures taken by owners to protect their dogs against the bites of sand flies in the focus area. Owners, advised by veterinarians, regularly treat dogs with topical insecticides and keep them indoors when sand flies are active. This could explain why there has been no increase in reported CanL cases in the past few decades. Health education is the first step to increase the benefits of an adequate One Health approach to control leishmaniosis as such an important zoonotic disease.

A strong link exists between canine *Leishmania* infection and HL [36, 37]. In the Madrid region, veterinarians have a good professional background to deal with CanL. However, at the time of the outbreak, for physicians working in a hypo-endemic area like Madrid, HL was a new disease presenting in their practices. It may also be true to say that HL diagnosis, mainly cutaneous forms, were probably underestimated before the outbreak. In fact, there was a reported high frequency of exposure to *L. infantum* among humans in Spain where 11.5–32.8% of schoolchildren and 44.2–52.8% of adults tested positive by the leishmanin skin test (LST) [36, 38]. Physicians may thus work together with veterinarians who have a longer track record of dealing with CanL. Looking for links between humans and animal patients and their similarities may be an interesting approach to expand our knowledge of this shared disease.

## Conclusions

The long-standing experience of veterinarians in managing leishmaniosis in the Madrid region and the health education they are giving to dog owners are key to the stable prevalence of this disease, despite the upsurge in HL cases seen in human medical practice. The role of health education (on the part of the owners, veterinarians, general practitioners, health authorities etc.) under the umbrella of One Health has been essential for the management of this outbreak which is currently in remission.

## References

[1] Arce A, Estirado A, Ordobás M, Sevilla S, García N, Moratilla L (2013). Re-emergence of leishmaniasis in Spain: community outbreak in Madrid, Spain, 2009 to 2012. Euro Surveill.

[2] Dantas-Torres F, Otranto D (2016). Best practices for preventing vector-borne diseases in dogs and humans. Trends Parasitol.

[3] Ready PD (2017). Managing the spread of canine leishmaniosis in Europe. Vet Rec.

[4] Millán J, Ferroglio E, Solano-Gallego L (2014). Role of wildlife in the epidemiology of *Leishmania infantum* infection in Europe. Parasitol Res.

[5] Baneth G, Koutinas AF, Solano-Gallego L, Bourdeau P, Ferrer L (2008). Canine leishmaniosis - new concepts and insights on an expanding zoonosis: part one. Trends Parasitol.

[6] Jiménez M, González E, Martín-Martín I, Hernández S, Molina R. Could wild rabbits (*Oryctolagus cuniculus*) be reservoirs for *Leishmania infantum* in the focus of Madrid, Spain? Vet Parasitol. 2014;202:296–300. 10.1016/j.vetpar.2014.03.027 24774435

[7] Molina R, Jiménez MI, Cruz I, Iriso A, Martín-Martín I, Sevillano O (2012). The hare (*Lepus granatensis*) as potential sylvatic reservoir of *Leishmania infantum* in Spain. Vet Parasitol.

[8] Montoya A, de Quadros LP, Mateo M, Hernández L, Gálvez R, Alcántara G, et al. *Leishmania infantum* infection in Bennett’s Wallabies (*Macropus rufogriseus rufogriseus*) in a Spanish wildlife park. J Zoo Wildl Med. 2016;47:586–93. 10.1638/2014-0216.1 27468032

[9] Lucientes Curdi J, Sánchez Acedo C, Castillo Hernández JA, Estrada PA (1988). Sobre la infección natural por L*eishmania* en *Phlebotomus perniciosus* Newstead, 1911 y *Phlebotomus ariasi* Tonnoir, 1921, en el foco de leishmaniosis de Zaragoza. Rev Iber Parasitol.

[10] Martín-Sánchez J, Guilvard E, Acedo-Sánchez C, Wolf-Echeverri M, Sanchís-Marín MC, Morillas-Márquez F (1994). *Phlebotomus perniciosus* Newstead, 1911, infection by various zymodemes of the *Leishmania infantum* complex in the Granada province (southern Spain). Int J Parasitol.

[11] Rioux JA, Guilvard E, Gállego J, Moreno G, Pratlong F, Portús M, IMEE (1986). *Phlebotomus ariasi* Tonnoir, 1921 et *Phlebotomus perniciosus* Newstead, 1911 vecteurs du complexe *Leishmania infantum* dans un même foyer: Infestations par deux zymodèmes syntopiques. A propos d’une enquête en Catalogne (Espagne). Leishmania Taxonomie et Phylogenèse Applications Éco-Épidemiologiques.

[12] Conesa Gallego E, Romera Lozano H, Martínez OE (1997). Estudio de las poblaciones de flebotomos (Diptera, Psychodidae) de la Comunidad de Madrid (España). An Biol.

[13] Gálvez R, Descalzo MA, Miró G, Jiménez MI, Martín O, Dos Santos-Brandao F (2010). Seasonal trends and spatial relations between environmental/meteorological factors and leishmaniosis sand fly vector abundances in Central Spain. Acta Trop.

[14] Gálvez R, Descalzo MA, Guerrero I, Miró G, Molina R. Mapping the current distribution and predicted spread of the leishmaniosis sand fly vector in the Madrid region (Spain) based on environmental variables and expected climate change. Vector Borne Zoon Dis. 2011;11:799–806. 10.1089/vbz.2010.0109 21417927

[15] Valcárcel Y, Bastero R, Anegón M, González S, Gil A (2008). Epidemiología de los ingresos hospitalarios por leishmaniasis en España (1999–2003)]. Enferm Infecc Microbiol Clin.

[16] Alvar J, Gutiérrez-Solar B, Molina R, López-Vélez R, García-Camacho A, Martínez P, et al. Prevalence of *Leishmania* infection among AIDS patients. Lancet. 1992;339:1427. 10.1016/0140-6736(92)91255-7 1350846

[17] López-Vélez R, Casado JL, Pintado V (2001). Decline of a visceral leishmaniasis epidemic in HIV-infected patients after the introduction of highly active antiretroviral therapy (HAART). Clin Microbiol Infect.

[18] Aránguez Ruiz E, Arce Arnáez A, Moratilla Monzo L, Estirado Gómez A, Iriso Calle A, De la Fuente US (2014). Análisis espacial de un brote de leishmaniasis en el sur del Área metropolitana de la Comunidad de Madrid. 2009–2013. Rev Salud Ambient.

[19] Jiménez M, González E, Iriso A, Marco E, Alegret A, Fuster F, Molina R (2013). Detection of *Leishmania infantum* and identification of blood meals in *Phlebotomus perniciosus* from a focus of human leishmaniasis in Madrid, Spain. Parasitol Res.

[20] Miró G, Rupérez C, Checa R, Gálvez R, Hernández L, García M (2014). Current status of *L. infantum* infection in stray cats in the Madrid region (Spain): implications for the recent outbreak of human leishmaniosis?. Parasit Vectors.

[21] Moreno I, Álvarez J, García N, de la Fuente S, Martínez I, Marino E, et al. Detection of anti-*Leishmania infantum* antibodies in sylvatic lagomorphs from an epidemic area of Madrid using the indirect immunofluorescence antibody test. Vet Parasitol. 2014;199:264–7. 10.1016/j.vetpar.2013.10.010 24211046

[22] Ayllon T, Tesouro MA, Amusátegui I, Villaescusa A, Rodríguez-Franco F, Sainz A (2008). Serologic and molecular evaluation of *Leishmania infantum* in cats from Central Spain. Ann NY Acad Sci.

[23] Ayllon T, Diniz PP, Breitschwerdt EB, Villaescusa A, Rodríguez-Franco F, Sainz A. Vector-borne diseases in client-owned and stray cats from Madrid, Spain. Vector Borne Zoon Dis. 2012;12:143–50. 10.1089/vbz.2011.0729 22022820

[24] Gálvez R, Miró G, Descalzo MA, Nieto J, Dado D, Martín O, Cubero E, Molina R (2010). Emerging trends in the seroprevalence of canine leishmaniosis in the Madrid region (central Spain). Vet Parasitol.

[25] Miró G, Montoya A, Mateo M, Alonso A, García S, García A (2007). A leishmaniosis surveillance system among stray dogs in the region of Madrid: ten years of serodiagnosis (1996–2006). Parasitol Res.

[26] Miró G, Montoya A, Roura X, Gálvez R, Sainz A (2013). Seropositivity rates for agents of canine vector-borne diseases in Spain: a multicentre study. Parasit Vectors.

[27] Patronek GJ, Beck AM, Glickman LT (1997). Dynamics of dog and cat populations in a community. J Am Vet Med Assoc.

[28] Scarlett JM, Miller L, Zawistowski S (2004). Pet population dynamics and animal shelter issues. Shelter medicine for veterinarians and staff.

[29] Mancianti F, Meciani N (1988). Specific serodiagnosis of canine leishmaniasis by indirect immunofluorescence, indirect hemagglutination, and counterimmunoelectrophoresis. Am J Vet Res.

[30] Gómez-Barroso D, Herrador Z, San Martín JV, Gherasim A, Aguado M, Romero-Mate A, et al. Spatial distribution and cluster analysis of a leishmaniasis outbreak in the south-western Madrid region, Spain, September 2009 to April 2013. Euro Surveill. 2015;20:11–20. 10.2807/1560-7917.es2015.20.7.21037 25719963

[31] Reisen WK (2010). Landscape epidemiology of vector-borne diseases. Annu Rev Entomol.

[32] Otranto D, Cantacessi C, Pfeffer M, Dantas-Torres F, Brianti E, Deplazes P (2015). The role of wild canids and felids in spreading parasites to dogs and cats in Europe. Part I: Protozoa and tick-borne agents. Vet Parasitol.

[33] García N, Moreno I, Álvarez J, de la Cruz ML, Navarro A, Pérez-Sancho M, et al. Evidence of *Leishmania infantum* infection in rabbits (*Oryctolagus cuniculus*) in a natural area in Madrid, Spain. Biomed Res Int. 2014;2014:318254. 10.1155/2014/318254 PMC3958779 24724079

[34] Solano-Gallego L, Miró G, Koutinas A, Cardoso L, Pennisi MG, Ferrer L (2011). LeishVet guidelines for the practical management of canine leishmaniosis. Parasit Vectors.

[35] Martín-Martín I, Molina R, Rohousova I, Drahota J, Volf P, Jiménez M (2014). High levels of anti-*Phlebotomus perniciosus* saliva antibodies in different vertebrate hosts from the re-emerging leishmaniosis focus in Madrid, Spain. Vet Parasitol.

[36] Acedo Sánchez C, Martin Sánchez J, Velez Bernal ID, Sanchís Marín MC, Louassini M, Maldonado JA, Morillas MF (1996). Leishmaniasis eco-epidemiology in the Alpujarra region (Granada Province, southern Spain). Int J Parasitol.

[37] Gavgani AS, Hodjati MH, Mohite H, Davies CR (2002). Effect of insecticide-impregnated dog collars on incidence of zoonotic visceral leishmaniasis in Iranian children: a matched-cluster randomised trial. Lancet.

[38] Moral L, Rubio EM, Moya M (2002). A leishmanin skin test survey in the human population of l’Alacanti region (Spain): implications for the epidemiology of *Leishmania infantum* infection in southern Europe. Trans R Soc Trop Med Hyg.

